# Neonatal phototherapy and cancer risk: a systematic review and meta-analysis

**DOI:** 10.3389/fped.2025.1667636

**Published:** 2025-11-24

**Authors:** Sloane J. Freeman, Charles D. G. Keown-Stoneman, Mariah Ghobrial, Thivia Jegathesan, Michael D. Sgro

**Affiliations:** 1 Women and Children’s Health Program, St. Michael’s Hospital, Unity Health Toronto, Toronto, ON, Canada; 2 Temerty Faculty of Medicine, University of Toronto, Toronto, ON, Canada; 3 Li Ka Shing Knowledge Institute, St. Michael’s Hospital, Unity Health Toronto, Toronto, ON, Canada; 4 Applied Health Research Centre, St. Michael’s Hospital, Unity Health Toronto, Toronto, ON, Canada; 5 Dalla Lana School of Public Health, University of Toronto, Toronto, ON, Canada

**Keywords:** phototherapy, hyperbilirubinemia, cancer, neonatology, systematic review, meta-analysis

## Abstract

**Objective:**

To evaluate the risk of cancer after phototherapy for neonatal hyperbilirubinemia.

**Study design:**

This was a systematic review and meta-analysis. Electronic databases, including PubMed, Embase, and Cochrane Library, were searched. Prospective and retrospective studies, case series, and review studies published between 1970 and 2025 were included. Studies underwent two stages of screening. The first phase was title and abstract screening. The second phase was a full-text review of studies deemed to meet the inclusion criteria. Risk of bias was assessed using ROBINS-E. Inverse-variance weighted multi-level random effects models were used for all analyses.

**Results:**

This systematic review and meta-analysis included 15 studies. Risk of bias was low in eight studies, one study was judged to have some concerns, and six studies were determined to have a high risk of bias. A total of 6,675,265 patient data points were included. Studies ranged from 1995–2022, with an age group from 35 weeks to 31 years old. Overall, there was an estimated 24% increased odds of cancer for those who received phototherapy compared to those who did not [OR = 1.24; 95% CI: (1.12, 1.36); *p* < 0.001].

**Conclusions:**

Phototherapy for neonatal hyperbilirubinemia was associated with a small increased risk of cancer up to age 31 years. This association must be balanced by the well-understood risk of Bilirubin-Induced Neurologic Dysfunction.

## Introduction

Phototherapy is the gold standard treatment for neonatal hyperbilirubinemia. In 2022, the American Academy of Pediatrics published revised clinical practice guidelines with increased thresholds for the use of phototherapy for term and near-term infants. Subsequently, a large US hospital network of 22,000 newborns reported a 47% decrease in phototherapy usage since the new guidelines were published, with 2% of newborns receiving phototherapy (1). European studies reported higher rates with close to 4% of neonates receiving phototherapy (2, 3). Phototherapy decreases serum levels of unconjugated bilirubin by changing the conformation of the bilirubin molecule to excretable water-soluble isomers. Without treatment, acute hyperbilirubinemia may lead to Bilirubin-Induced Neurologic Dysfunction (BIND). BIND is the constellation of neurologic signs and symptoms that include acute bilirubin encephalopathy and may result in the sequelae of chronic bilirubin encephalopathy, hearing loss, and visuo-oculomotor disturbances (4–6). Animal and cell culture studies have identified concerns around phototherapy-induced DNA damage, raising questions around a potential cancer risk (7–18). However, its potential as a carcinogen has been investigated in numerous observational studies with mixed results (19–22).

Previous meta-analyses evaluating cancer risk after exposure to phototherapy in the neonatal period have shown a small positive association (23–26), however these meta-analyses analyzed fewer studies than the present one, either because they separated cohort and case control studies (23, 24), or excluded studies included within their systematic reviews from their primary analyses without sufficient explaination (25, 26). The aim of our study was to perform an updated, inclusive, and focused systematic review and meta-analysis evaluating the association between phototherapy in the neonatal period and cancer risk, excluding benign and dysplastic nevi, using weighted analysis, and including only studies with appropriate tests of association or sufficient detail for re-analysis, to increase the reliability of our findings.

## Methods

### Study design

This is a systematic review and meta-analysis which followed The Cochrane Handbook for Systematic Reviews (27) and the PRISMA 2020 reporting guidelines for reporting Systematic Reviews and Meta-Analyses (28).

### Search process

A literature search of electronic databases including: Medline, Embase, Cochrane Central, CINAHL, and Scopus was performed on March 28, 2022 and June 18, 2025. The search strategy underwent a review process in accordance with the Peer Review for Electronic Search Strategies (PRESS) guidelines (29). The search terms and keywords used covered three main concepts, “Phototherapy,” “Neonates,” and “Cancer,” including any of these concepts' synonymous words. The full search strategy can be found in the Supplementary Data.

### Inclusion criteria

We included human studies published in English between 1970 and 2025, with neonates of all gestational ages who underwent phototherapy within 28 days of age. Peer-reviewed observational studies, including prospective and retrospective studies, case series studies, and review studies were included.

### Exclusion criteria

Studies were excluded if they were theoretical studies based on human cells, *in vitro* and/or animal studies, studies where phototherapy was the outcome variable, studies where the outcome was not the impact of phototherapy, studies where the outcomes were benign and/or dysplastic nevi, or studies which did not report the side effects of phototherapy. Additionally, one study was excluded because it represented a duplicate sample from another included study.

### Outcome

Our primary outcome was overall cancer risk.

### Data extraction

Studies underwent two stages of screening. The first phase was title/abstract screening. The second phase was a full-text review of studies deemed to meet the inclusion criteria. Two independent reviewers were assigned to each phase. Each reviewer independently screened studies, determined the eligibility of papers and decided on inclusion status, with two additional independent reviewers resolving any conflicts among the initial reviewers' decisions. Records and data were managed throughout the review using Covidence (30), a systematic review software, and EndNote (31), a bibliographic software.

### Risk of bias assessment

Risk of bias was assessed using Risk of Bias in Non-randomized Studies of Exposures (ROBINS-E) (32). Risk of bias was assessed for seven domains, including: confounding, measurement of the exposure, selection of participants into the study (or into the analysis), post-exposure interventions, missing data, measurement of the outcome, and selection of the reported results (32). Two independent reviewers completed the assessment for each study. The overall score was reported as low, some concerns, or high risk of bias.

### Statistical analysis

Inverse-variance weighted multi-level random effects models were used for all analyses. Weights were assigned using the inverse of an effect size's variance (i.e., the squared standard error) to determine the strength of evidence within each study. For example, larger studies with less variance in their estimates tend to contribute more weight in the meta-analyses. These models account for heterogeneity between estimates from different study designs, with random effects explicitly modelled for each individual study in the analyses. For the overall cancer risk analyses, an additional random effect was included for heterogeneity from the type of cancers (“skin”, “blood”, “solid organ”, and “any or other”). Results were pooled on the odds ratio (OR) scale. For studies that reported other metrics, such as standardized incidence ratio estimates, the equivalent OR was estimated from the reported data. For studies with zero cancers in one of the cancer adjusted ORs and standard errors were estimated. Egger's tests (33) were performed, and funnel plots were produced for each outcome to assess potential publication bias. All meta-analyses and re-estimation of individual study effects were performed using the metafor package (34) in R version 4.4.2 (35).

## Results

**Figure 1:**
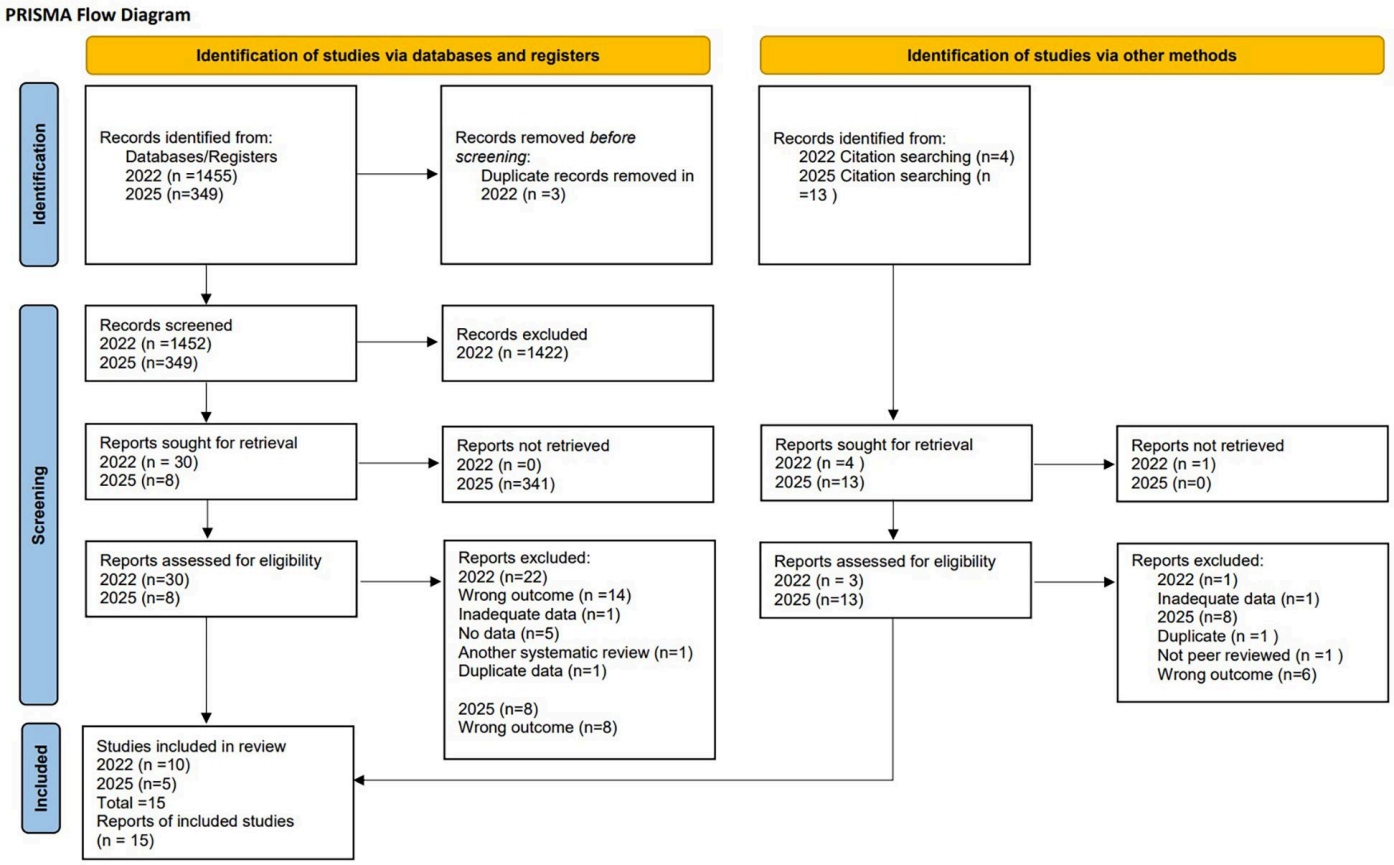
PRISMA flow diagram.

Our systematic review and meta-analysis included 15 studies (19–22, 36–46). See PRISMA flow diagram, Figure 1. A total of 6,675,265 patient data points were included in the analysis through a combination of case-control and retrospective cohort studies. Included studies ranged from 1995–2022, with an age group from 35 weeks to 31 years old, Table 1.

**Table 1:** Study characteristics.

Author, Year, Country	Study Design	Sample Size	Effect estimates	Age at Diagnosis	Follow up Period	Outcomes	ROBINS-E Overall Score Indicating Level of Risk of Bias	Control for Confounding Variables^a^	Exclusions
Auger et al., Canada (19)	Cohort	786,998	Incidence ratios, hazard ratios	Not reported	11 years	Any cancer: aHR 1.34 (95% CI 0.99–1.83), any solid tumor: aHR 1.36 (95% CI 0.91–2.02), any hematopoietic cancer: aHR 1.32 (95% CI 0.81–2.14)	Low	Yes. Confounding variables included: maternal age, gestational age, birth weight, multiple births, c-section, sex, congenital anomalies (Down syndrome, other chromosomal anomalies), socioeconomic deprivation, place of residence, and birth year.	None
		Exposed = 32,314 Unexposed = 754,684							
Brewster et al., Scotland (36)	Cohort	77,518	Incidence ratios	Median 24.3 years (range, 15–29 years)	Maximum 30 years	Melanoma: IR 1.40 (95% CI 0.17–5.04), Squamous cell carcinoma: IR -, Basal cell carcinoma: IR 0.00 (95% CI 0.00–3.11)	High	No control for confounding variables.	None
		Exposed = 5,868 Unexposed = 71,650							
Bugaiski-Shaked et al., Israel (20)	Cohort	342,172 Exposed = 18,797 Unexposed = 323,375	Hazard ratios	Exposed = Mean 6.15 ± 5.9 years, Un-exposed = mean 8.38 ± 6.6 years	Median = 9.5 years (range, birth to 18 years	All cancer: aHR 1.33 (95% CI 0.95–1.84), Solid tumors: aHR 1.02 (95% CI 0.62–1.69), hematopoietic malignancies leukemia, lymphoma): aHR 1.51 (95% CI 0.99–2.29)	Low	Yes. Confounding variables included: preterm birth, maternal age	Excluded newborns with multiple gestations, prematurity, and malformations from the analysis.
Cnattingius et al., Sweden (37)	Case-control	Case = 98 Control = 490	OR	<2 yr. 16% 2–4 yr. 53% > 5 yr: 31%	Maximum 19 years	Myeloid leukemia: OR 4.3 (95% CI 0.9–21.9)	High	No control for confounding variables.	None
Cnattingius et al., Sweden (38)	Case-control	3,678	OR	39% (*n* = 38)= < 2 years	Maximum 19 years	Myeloid leukemia: OR 1.0 (95% CI 0.5–1.8)	High	No control for confounding variables.	None
		Case = 613							
		Control = 3,065		30% (*n* = 29) = 2–4 years					
				21% (21) = 5–9 years					
				10% = >10 years					
Digitale et al., United States (21)	Cohort	139 100	Hazard ratios	Not reported	Maximum 24 years	Any cancer: aHR 1.13 (95% CI 0.83–1.54), any hematopoietic cancer: aHR 1.17 (95% CI 0.74–1.83), any solid tumor: aHR 1.01 (95% CI 0.65–1.58)	Low	Yes. Confounding variables included: sex, race and/or ethnicity gestational age, delivery mode, facility of birth, year of birth, maternal age, multiple birth, birth weight, Down syndrome, congenital anomalies, chromosomal abnormalities other than Down syndrome, early hyperbilirubinemia, and TSB level in relation to the phototherapy threshold.	Excluded Down syndrome from propensity models for hematopoietic cancers.
		Exposed- = 42,266 Unexposed = 96,834							
Kadivar et al., Iran (39)	Case Control	Case = 500	OR	Case = mean 6.86 ± 4.02 years Control = 4.39 ± 2.53 years	Maximum 14 years	Any cancer: HR 0.71 (95% CI 0.54–0.95)	Some concerns.	Yes. Confounding variables included: gender, maternal age during pregnancy, and paternal smoking, and birth weight.	Excluded children over 14 years of age, children with Down syndrome, Von Hippel-Lindau, Beckwith-Wiedemann, and Neurofibromatosis syndromes, and other congenital major anomalies. Children with incomplete data or permission were also excluded.
		Control = 500							
Ku et al., Taiwan (40)	Cohort	Exposed = 4,744	Incident rate ratios	Not reported	Maximum 5 years	Skin cancer: prevalence in exposed group: 0.02% (1 case), non-exposed icteric group: 0% (0 case), and non-exposed non-icteric group 0.01% (15 cases)	Low	Yes. Confounding variables included: socioeconomic status, urbanization, special healthcare needs, congenital skin disease, infection, sex, preterm, low birth weight, high birth weight, and other or maternal conditions such as birth asphyxia, placental complications.	None
		Unexposed = 112,297							
						Total cancer prevalence: exposed group 0.88% (42 cases), non-exposed icteric group: 0.84% (42 cases), and non-exposed non-icteric group: 0.74% (795 cases)			
Linet et al., Sweden (41)	Case-control	3,420	OR	118 (20.7%) = <2 years	Maximum 16 years	Brain tumors: aOR 1.3 (95% CI 0.7–2.5)	Some concerns	Yes. Control for confounding variables, but not specified.	None
		Case = 570							
				159 (27.9%) = 2–4 years					
		Control = 2,850							
				179 (31.4%) = 5–9 years					
				114 (20.0%) = >10 years					
Olsen et al., Denmark (42)	Cohort	55,120	Standardized incidence ratios	Not reported	Mean 9.1 years (range, 0–15 years)	All cancers: SIR 1.0 (95% CI 0.8–1.3), leukemia: SIR 1.2 (95% CI 0.8–1.7)	High	No control for confounding.	Excluded premature neonates and neonates with hemolytic disease form the analysis.
		Exposed = 55,120 Unexposed = 668,070							
Podvin et al., United States (43)	Case-control	6,545	OR	Not reported	Maximum 19 years	Leukemia: aOR 2.2 (9%% CI 1.0–4.9)	Low	Yes. Control for confounding variables included: race/ethnicity, birthweight, gender, gestational age, maternal and paternal age, maternal smoking, maternal diabetes, maternal marital status, and parity.	None
		Case = 595							
		Control = 5,950							
Roman et al., United Kingdom (44)	Case-control	429	OR	3 months to 29 years	Maximum 31 years	Leukemia: OR 0.5 (95% CI 0.1–2.3), Non-Hodgkins Lymphoma OR oo (95% CI 0.3- oo)	High	No control for confounding.	Newborns from multiple pregnancy were ineligible. Excluded newborns with identifiable chromosomal anomalies or other severe malformations (e.g., spina bifida) from the analysis.
		Case = 177							
		Control = 354							
Sabzevari et al., Iran (45)	Case-control	232	OR	24 (20.7%) = 1–2 years	Maximum 4 years	Any cancer: aOR 1.38 (95% CI 0.03–55.53)	Low	Yes. Confounding variables included age, gender, maternal age, maternal addiction, neonatal nutrition, and urban or rural life, maternal infection and maternal educational level.	Children over 4 years old, children with syndromes related to childhood malignancies including Down, Von Hippel-Lindau, Beckwith-Wiedemann, and Neurofbromatosis syndromes, major congenital anomalies, prematurity, incomplete or doubtful data, Children with a history of blood exchange during the neonatal period were excluded.
		Case = 116							
				37 (31.9%) = 2–3 years					
		Control = 116							
				55 (47.4%) = 3–4 years					
Seppala et al., Finland (22)	Case-control	2,029	OR	Not reported	Maximum 18 years	Any cancer: aOR 1.11 (95% CI 0.91–1.35)	Low	Yes. Confounding variables included: birth weight for gestational age, and birth weight alone, maternal age, parity, and maternal smoking during pregnancy.	None
		Case: 2,029							
		Control: 10,103							
Wickremasinghe et al., United States (46)	Cohort	5,144,861	Incidence ratios, OR, risk ratios	2 to 12 months	Maximum 1 year	All cancer: Risk difference: 11.6 (9%% CI 3.6–20.7), All cancer: propensity aOR 1.4 (1.1–1.9)	Low	Yes. Confounding variable sincluded: gender, birth weight, gestational age, large for gestational age, twin birth, cesarean delivery, payer source, year of birth, maternal race, paternal race, maternal Hispanic ethnicity, maternal age, paternal age, maternal education, paternal education, Down syndrome, other chromosomal anomalies, and non-chromosomal congenital anomalies.	Exlcuded neonates with Down syndrome from the primary analysis.
		Exposed = 178,017							
		Unexposed = 4,966,832							

a Using Robins-E: appropriate methods to control for confounders measured at baseline include stratification, regression, matching, standardization, and inverse probability weighting. The analysis may control for individual variables or for estimated propensity scores (inverse probability weighting is based on a function of the propensity score) (32).

### Risk of bias assessment

Using ROBINS-E, eight studies were determined to have a low risk of bias, one study was judged to have some concerns, and six studies were determined to have a high risk of bias based on lack of control for confounding factors (the other domains were assessed as low risk of bias for all studies). Important confounding factors were those where adjustment was expected to lead to an important change in the estimated effect of the exposure (32) (Table 1).

### Any cancer risk

**Figure 2:**
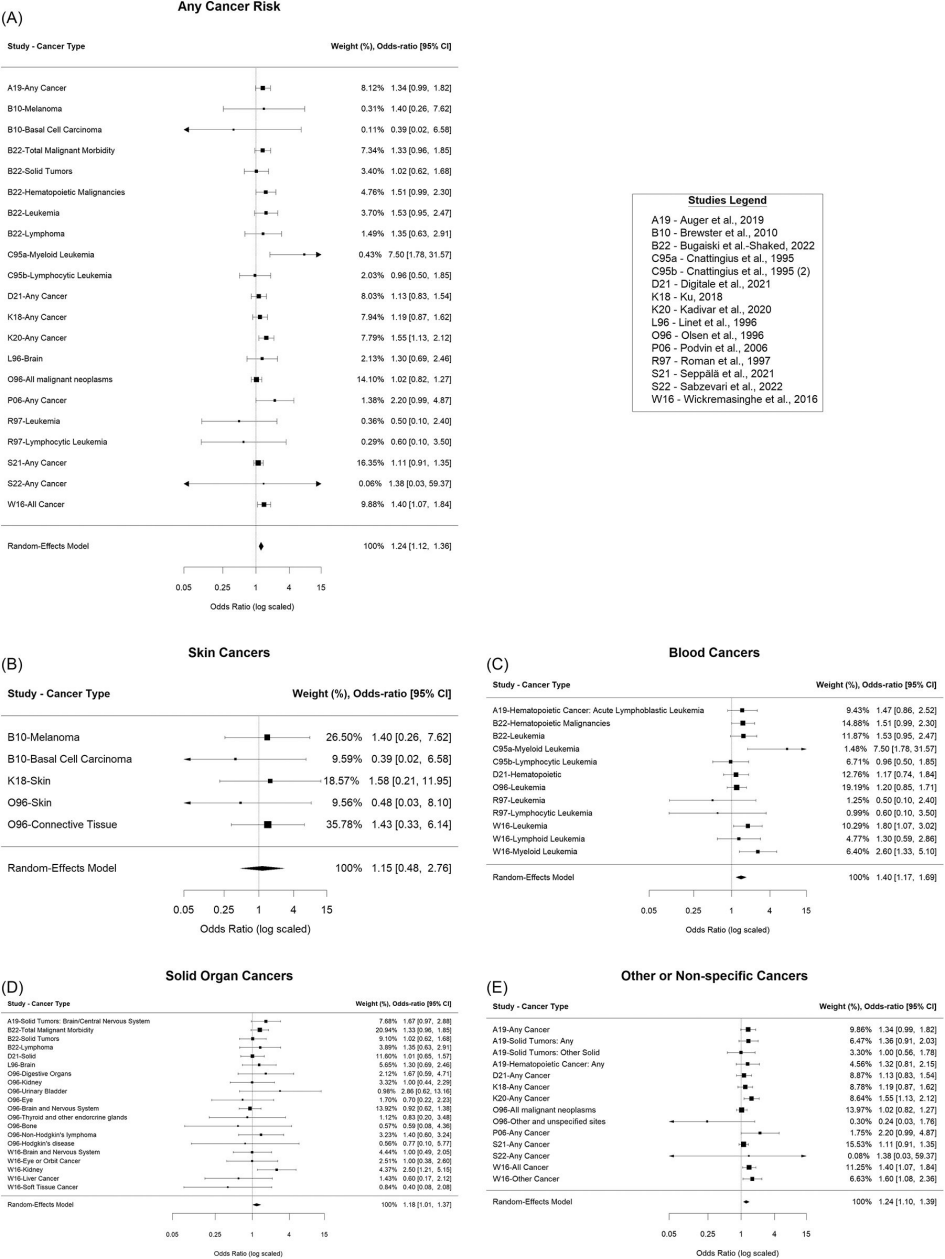
Forest plots. **(A)** Overall cancer risk, **(B)** Skin cancer risk, **(C)** Blood cancer risk, **(D)** Solid organ cancer risk, **(E)** Other or non-specific cancer risk.

**Figure 3:**
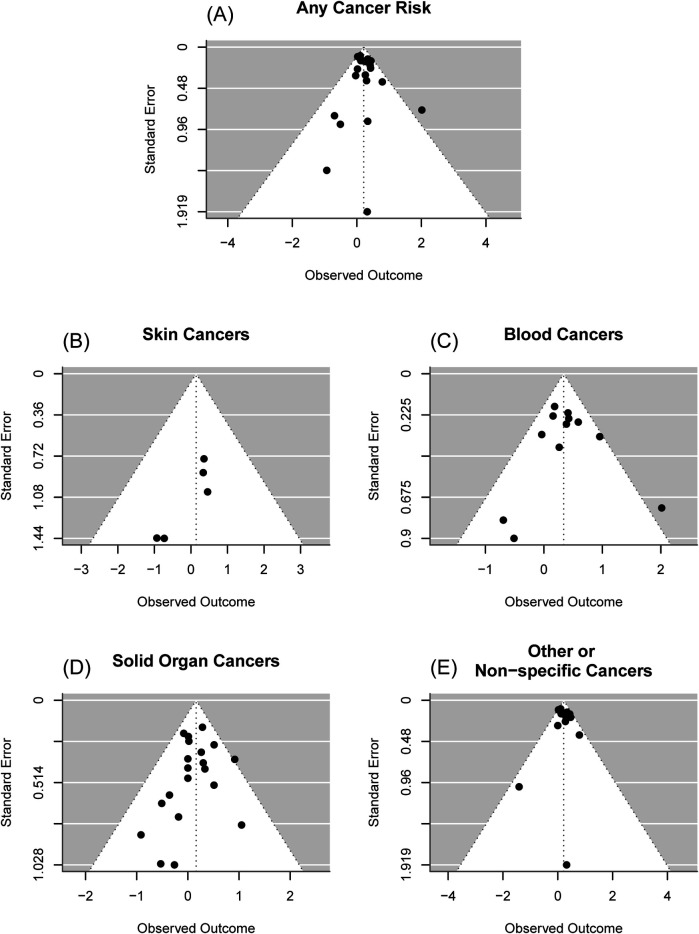
Funnel plots. **(A)** Overall cancer risk. From an Egger's test, there was insufficient evidence of publication bias in reporting of overall cancer risk (*p* = 0.98), **(B)** Skin cancer risk. From an Egger's test, there was insufficient evidence of publication bias in reporting of skin cancer risk (*p* = 0.34), **(C)** Blood cancer risk, From an Egger's test, there was insufficient evidence of publication bias in reporting of blood cancer risk (*p* = 0.73), **(D)** Solid organ cancer risk. From an Egger's test, there was insufficient evidence of publication bias in reporting of solid organ cancer risk (*p* = 0.49), **(E)** Other or non-specific cancer risk. From an Egger's test, there was insufficient evidence of publication bias in reporting of other or non-specific cancer risk (*p* = 0.73).

Overall, there was an estimated 24% increased odds of cancer for those who received phototherapy compared to those who did not [OR = 1.24; 95% confidence interval (CI): (1.12, 1.36); *p* < 0.001], Figure 2A. It was estimated that 9.33% of the total variance was due to heterogeneity (I^2^ = 8.72%). There was insufficient evidence of publication bias in the reporting of any cancer risk (*p* = 0.45), Figure 3A. Assuming a baseline risk of cancer of (1/463) (47), this estimate would lead to an approximate expected additional 51 cancer diagnoses per 100,000 children treated with phototherapy. This example baseline risk of cancer was taken from Auger et al. (19) and represents the baseline risk over the first 11 years of age.

### Skin cancer risk

For skin cancers, there was insufficient evidence of a difference in the odds of cancer for those who received phototherapy compared to those who did not [OR = 1.15; 95% CI: (0.48, 2.76); *p* = 0.75], Figure 2B. It was estimated that 0.00% of the total variance was due to heterogeneity (I^2^ = 0.00%). There was insufficient evidence of publication bias in the reporting of skin cancer risk (*p* = 0.34), Figure 3B.

### Blood cancer risk

For cancers of the blood, there was an estimated 40% increased odds of cancer for those who received phototherapy compared to those who did not [OR = 1.40; 95% CI: (1.17, 1.69); *p* < 0.001], Figure 2C. It was estimated that 10.36% of the total variance was due to heterogeneity (I^2^ = 10.36%). There was insufficient evidence of publication bias in the reporting of blood cancer risk (*p* = 0.73), Figure 3C.

### Solid organ cancer risk

For solid organ cancers, there was an estimated 18% increased odds of cancer for those who received phototherapy compared to those who did not [OR = 1.18; 95% CI: (1.01, 1.37); *p* = 0.04], Figure 2D. It was estimated that 0.00% of the total variance was due to heterogeneity (I^2^ = 0.00%). There was insufficient evidence of publication bias in the reporting of solid organ cancer risk (*p* = 0.49), Figure 3D.

### Other or non-specific cancer risk

For other or non-specific cancers, there was an estimated 24% increased odds of cancer for those who received phototherapy compared to those who did not [OR = 1.24; 95% CI: (1.10, 1.39); *p* < 0.001], Figure 2E. It was estimated that 23.44% of the total variance was due to heterogeneity (I^2^ = 23.44%). There was insufficient evidence of publication bias in reporting of any or other cancer risk (*p* = 0.73), Figure 3E.

In addition to I^2^, the measure of variance due to overall heterogeneity, we also estimated *τ*^2^, the estimate of between-study variance from the random effects (34). For all reported meta-analyses, the *τ*^2^ estimate was 0.00.

## Discussion

In this systematic review and meta-analysis of 15 studies and 6,675,265 children, phototherapy for neonatal hyperbilirubinemia was associated with a small increased risk of cancer up to age 31 years (24% increased odds). Our findings are similar to four previous systematic reviews and meta-analyses (23–26); however, there were differences in our methodological approach. The previous systematic reviews and meta-analyses either separated their analyses based on study design (case-control or cohort) (23, 24), or excluded studies from their primary analyses for other reasons (25, 26). We adopted an inclusive approach, pooling all 15 studies into one meta-analysis. Since cancer is a rare outcome, and because relative risks, odds ratios, and other multiplicative effect estimates approximate each other for rare outcomes, we combined estimates from various study designs (case-control or cohort) and analyzed them together. However, we included modelling to account for variance from each study given the different study types and multiple estimates coming from individual studies. Another distinction from three of the four previous meta-analyses evaluating childhood cancer risk (23, 24, 26), is that our study evaluated cancer risk into adulthood. Other differences were that we included only peer-reviewed studies, excluded studies which did not report findings with sufficient detail to obtain or perform appropriate tests of association (48), and excluded studies with duplicate cases [two studies found in our systematic review, Newman et al. (49) and Digitale et al. (21, 49), reported on the same cohort, therefore we only included the later study by Digitale et al.].

In terms of specific cancer types, blood cancers and solid organ cancers were found to have the greatest association with cancer risk, with an estimated 40% and 18% increased odds of cancer, respectively, for those who received phototherapy compared to those who did not. This is consistent with previous meta-analyses, which showed that blood cancers had the greatest association with cancer risk (23–26).

There are several plausible mechanisms for the carcinogenic effects of phototherapy. Phototherapy generates oxygen radicals, which can lead to DNA damage (14). Studies have demonstrated DNA damage and cytokine changes among term neonates, and evidence of oxidative stress among premature neonates after phototherapy (11, 13, 16). Phototherapy may also induce apoptosis and DNA damage specifically in lymphocytes, potentially affecting hematopoiesis and contributing to blood cancer risk (50). Studies have also shown a positive association between the duration of phototherapy and markers of DNA damage (14). DNA damage over time can potentially lead to carcinogenic gene modifications. Similarly, studies have shown that higher intensity light causes more DNA damage than conventional light therapy (51). However, a recent study demonstrated that the negative effect of phototherapy on sister chromatid exchange frequency (an index of genomic stability in response to environmental or genetic mutagens) was temporary. After 3.5 years of follow-up, differences in mean sister chromatid exchange values had disappeared between the majority of children who received phototherapy in the neonatal period and healthy children who had not received phototherapy (52).

It should be noted that several studies included in our meta-analysis did not adjust for confounding variables such as hyperbilirubinemia, prematurity, intrauterine growth restriction, congenital anomalies, as well as maternal factors such as gestational diabetes, all of which may increase cancer risk, as well as the need for phototherapy, and must be considered when interpreting our findings. Adjustment for hyperbilirubinemia may have been avoided in some studies, as it too was collinear with the phototherapy exposure; however, this remains a limitation as there is evidence that hyperbilirubinemia is a confounder that is independently associated with cancer risk (19), along with its more obvious connection to the decision to use phototherapy. The association between hyperbilirubinemia and cancer risk deserves additional attention. A large cohort study and a case-control study included in our meta-analysis evaluated hyperbilirubinemia and cancer risk and demonstrated a small increased risk of cancer (19, 43). Studies have also shown a positive association between hyperbilirubinemia and DNA damage, as well as enhanced apoptosis among circulating lymphocytes of term infants (53).

Other factors that were not addressed in our meta-analysis were the duration and method of phototherapy delivery. It may be postulated that longer durations and greater intensity of light could influence the risk of malignancy (14, 51). These factors were not reported in the majority of studies included in this meta-analysis or previous meta-analyses and therefore could not be assessed.

While our results show an association between phototherapy and cancer, the clinical significance remains unclear. An OR of 1.24 indicates an added 24% increase in the odds of developing cancer after phototherapy exposure; however, since the baseline risk of childhood cancer is very low, phototherapy would lead to a small number of additional cancer diagnoses (e.g., 51/100,000 children treated with phototherapy), some of which may be due to increased risk due to hyperbilirubinemia. This low risk must be balanced with the risk of untreated hyperbilirubinemia. Untreated hyperbilirubinemia in the neonatal period can lead to BIND with devastating neurodevelopmental consequences (4–6). Current phototherapy guidelines (1, 57) are associated with a very low incidence of neurodevelopmental complications from hyperbilirubinemia, with approximately 1 in every 50,000–100,000 children developing chronic bilirubin encephalopathy (58). Historic data demonstrate that higher thresholds for initiating phototherapy and a lack of routine screening for hyperbilirubinemia in the 1990s (59) led to an increased risk of both acute and chronic bilirubin encephalopathy in Canada (58, 60). However, administrative data from the California Department of Developmental Services did not support a resurgence in kernicterus. Trends in acute bilirubin encephalopathy were not reported (61). There is clear evidence that prior to the use of phototherapy for hyperbilirubinemia, neurodevelopmental consequences were much more common, and are still seen in developing countries that have reduced access to bilirubin monitoring and phototherapy (62, 63). While recent meta-analyses including this one show a very small but reproducible risk of cancer after phototherapy, this association must be balanced by the well-understood risk of BIND. This study highlights the importance of adhering to phototherapy guidelines, including close serum bilirubin monitoring of children at risk of severe hyperbilirubinemia, and the appropriate use of phototherapy.

## Limitations

This study had some limitations. First, several studies included in our meta-analysis did not control for premature births and other confounding factors. Furthermore, premature neonates may be affected differently by phototherapy as they have distinct physiology from term infants, as well as thinner skin, which may be more vulnerable to the potential mutagenic effects of phototherapy (54, 55). Premature infants are also more likely to require a longer duration of phototherapy compared to term infants with hyperbilirubinemia, which may increase the risk of cancer. Second, only observational studies (case-control and cohort studies) were included in our meta-analysis, and therefore, we cannot make any definitive conclusions about a causal relationship between phototherapy and cancer. Third, the high degree of heterogeneity across individual study designs included in our meta-analysis made interpretation more challenging. Furthermore, the variability in follow-up periods among studies limited our ability to analyze the age at cancer diagnosis. However, the heterogeneity among included studies improved the generalizability of our findings (56). Finally, two studies included in our meta-analysis (Wickramasinghe et al. and Digitale et al.) (21, 46) were conducted in the same state with overlapping study periods. While data was extracted from different administrative databases, there may have been an overlap between their samples.

## Conclusions

We found a small increased risk of cancer up to age 31 years (24% increased odds) after neonatal phototherapy. Further research is needed to explore the risk of cancer after phototherapy, adjusting for confounding factors, as well as the potential impacts of phototherapy duration and intensity. Additionally, more research is needed to evaluate the effects of phototherapy in the very premature infant.

## Data Availability

The raw data supporting the conclusions of this article will be made available by the authors, without undue reservation.
