# Supplementary material for: Neonatal phototherapy and cancer risk: a systematic review and meta-analysis
Source: Front Pediatr. 2025 Nov 24;13:1667636. doi: 10.3389/fped.2025.1667636 (PMC12682806; doi:10.3389/fped.2025.1667636)
Supplement: Supplementary file 1 [file Datasheet1.docx]

**Search Strategy**

**March 28^th^ 2022**

The search strategy terms and keywords used in searching the database are found below.

**Search Filters**

The Ovid search subset for 'cancer' has been applied where available. The full search filter can be found here:<https://www.nlm.nih.gov/bsd/pubmed_subsets/cancer_strategy.html>.

**Number of results per database**

| **Databases Searched** | **Date of Search** | **Number of Results** |
| --- | --- | --- |
| All Ovid Medline <1946 - present> | March 28, 2022 | 612 |
| EBM Reviews - Cochrane Central Register of Controlled Trials <January 2022> | March 28, 2022 | 9 |
| CINAHL Complete (EBSCO) | March 28, 2022 | 51 |
| Embase Classic+Embase <1947 to 2022 March 25> | March 28, 2022 | 878 |
| Scopus (Elsevier) | March 28, 2022 | 115 |
|  | Total | 1665 |
|  | **Total**  **(After deduplication)** | **1455** |

**Search Strategies**

**Medline**

1 exp Infant/ 1212223

2 exp Hyperbilirubinemia, Neonatal/ 7608

3 neonat*.tw,kf. 294741

4 (infant* or infanc*).tw,kf. 525185

5 newborn*.tw,kf. 188554

6 1 or 2 or 3 or 4 or 5 1521448

7 Phototherapy/ 10571

8 phototherap*.tw,kf. 11052

9 light therap*.tw,kf. 2573

10 blue light.tw,kf. 10569

11 7 or 8 or 9 or 10 29177

12 6 and 11 3549

13 limit 12 to cancer 616

14 limit 13 to yr="1970 -Current" 612

<https://ovidsp.ovid.com/ovidweb.cgi?T=JS&NEWS=N&PAGE=main&SHAREDSEARCHID=Ynu54IJGpbkZ2VGMMoUDIlSXLz4A4HEX0TSz27BVdaoi0FnunHLF1uE91diNSURR>

**CINAHL**

| S16 | S6 AND S11 AND S15 | View Results (51) |
| --- | --- | --- |
|  |  |  |
|  |  |  |
| S15 | S12 OR S13 OR S14 | View Results (885,167) |
|  |  |  |
|  |  |  |
| S14 | TI ( (cancer* or neoplas* or carcino* or adenocarcinoma* or malignanc* or tumor* or tumour* or malignan* or metasta* or sarcoma* or chromatid exchange* or leukemia* or leukaemia* or melanoma* or biops*) ) OR AB ( (cancer* or neoplas* or carcino* or adenocarcinoma* or malignanc* or tumor* or tumour* or malignan* or metasta* or sarcoma* or chromatid exchange* or leukemia* or leukaemia* or melanoma* or biops*) ) | View Results (718,969) |
|  |  |  |
|  |  |  |
| S13 | (MH "Biopsy+") | View Results (50,891) |
|  |  |  |
|  |  |  |
| S12 | (MH "Neoplasms+") | View Results (621,727) |
|  |  |  |
|  |  |  |
| S11 | S7 OR S8 OR S9 OR S10 | View Results (7,551) |
|  |  |  |
|  |  |  |
| S10 | TI "blue light" OR AB "blue light" | View Results (585) |
|  |  |  |
|  |  |  |
| S9 | TI "light therap*" OR AB "light therap*" | View Results (750) |
|  |  |  |
|  |  |  |
| S8 | TI phototherap* OR AB phototherap* | View Results (1,941) |
|  |  |  |
|  |  |  |
| S7 | (MH "Phototherapy+") | View Results (5,993) |
|  |  |  |
|  |  |  |
| S6 | S1 OR S2 OR S3 OR S4 OR S5 | View Results (354,648) |
|  |  |  |
|  |  |  |
| S5 | TI newborn* OR AB newborn* | View Results (35,018) |
|  |  |  |
|  |  |  |
| S4 | TI ( (infant* or infanc*) ) OR AB ( (infant* or infanc*) ) | View Results (127,026) |
|  |  |  |
|  |  |  |
| S3 | TI neonat* OR AB neonat* | View Results (76,506) |
|  |  |  |
|  |  |  |
| S2 | (MH "Hyperbilirubinemia, Neonatal+") | View Results (1,687) |
|  |  |  |
|  |  |  |
| S1 | (MH "Infant+") | View Results (281,090) |

**Embase**

1 exp infant/ 1242914

2 neonatal hyperbilirubinemia/ 1294

3 neonat*.tw,kf. 404361

4 (infant* or infanc*).tw,kf. 642243

5 newborn*.tw,kf. 249027

6 1 or 2 or 3 or 4 or 5 1632305

7 exp phototherapy/ 103242

8 exp phototherapy device/ 1808

9 phototherap*.tw,kf. 15667

10 light therap*.tw,kf. 3636

11 "blue light".tw,kf. 11603

12 7 or 8 or 9 or 10 or 11 117574

13 exp neoplasm/ 5407487

14 exp biopsy/ 883393

15 (cancer* or neoplas* or carcino* or adenocarcinoma* or malignanc* or tumor* or tumour* or malignan* or metasta* or sarcoma* or chromatid exchange* or leukemia* or leukaemia* or melanoma* or biops*).tw,kf. 6056998

16 13 or 14 or 15 7265822

17 6 and 12 and 16 1131

18 limit 17 to embase 882

19 limit 18 to yr="1970 -Current" 878

<https://myaccess.library.utoronto.ca/login?url=http://ovidsp.ovid.com/ovidweb.cgi?T=JS&NEWS=N&PAGE=main&SHAREDSEARCHID=2yWKXD04kcMR4dEjFIqrZpquHSoC3uffe9G7vqJva2YC6xlhVK54zPeAOYdALWRbp>

**Scopus**

( ( TITLE-ABS-KEY ( neonat* ) ) OR ( TITLE-ABS-KEY ( ( infant* OR infanc* ) ) ) OR ( TITLE-ABS-KEY ( newborn* ) ) ) AND ( ( TITLE-ABS-KEY ( phototherap* ) ) OR ( TITLE-ABS-KEY ( "light therap*" ) ) OR ( TITLE-ABS-KEY ( "blue light" ) ) ) AND ( TITLE-ABS-KEY ( cancer* OR neoplas* OR carcino* OR adenocarcinoma* OR malignanc* OR tumor* OR tumour* OR malignan* OR metasta* OR sarcoma* OR chromatid AND exchange* OR leukemia* OR leukaemia* OR melanoma* OR biops* ) ) AND ( EXCLUDE ( PUBYEAR , 1967 ) OR EXCLUDE ( PUBYEAR , 1966 ) )

**June 18^th^ 2025**

The search strategy takes the basic format of: Neonates AND Phototherapy AND Cancer**. This update is designed to capture results from this search from the original search run date, March 28, 2022, to present.**

**Limits**

A publication date limit has been applied to the search (1970-present). **For the update, entry date limits have been added to capture just those records added to the databases from the original search run date to present.**

**Search Filters**

The Ovid search subset for 'cancer' has been applied where available. The full search filter can be found here: <https://www.nlm.nih.gov/bsd/pubmed_subsets/cancer_strategy.html>.

**Number of Results Per Database**

| **Databases Searched** | **Date of Search** | **Number of Results** |
| --- | --- | --- |
| All Ovid Medline <1946 - present> | June 17, 2025 | 75 |
| EBM Reviews - Cochrane Central Register of Controlled Trials <May 2025> | June 17, 2025 | 2 |
| CINAHL Complete (EBSCO) | June 17, 2025 | 10 |
| Embase Classic+Embase <1947 to 2025 June 16> | June 17, 2025 | 278 |
| Scopus (Elsevier) | June 17, 2025 | 30 |
| Total | 395 |  |
| **Total**  **(After deduplication)** | **349** |  |

**Search Strategies**

**Medline**

1 exp Infant/ 1312391

2 exp Hyperbilirubinemia, Neonatal/ 8071

3 neonat*.tw,kf. 343898

4 (infant* or infanc*).tw,kf. 592207

5 newborn*.tw,kf. 211818

6 1 or 2 or 3 or 4 or 5 [Neonates search string] 1670986

7 Phototherapy/ 12949

8 phototherap*.tw,kf. 14351

9 light therap*.tw,kf. 3547

10 blue light.tw,kf. 13950

11 7 or 8 or 9 or 10 [Phototherapy search string] 37682

12 6 and 11 [Overall results for references on neonates and phototherapy] 4072

13 limit 12 to cancer [Ovid search subset for 'cancer'. Full search filter can be found here: https://www.nlm.nih.gov/bsd/pubmed_subsets/cancer_strategy.html] 686

14 limit 13 to yr="1970 -Current" 682

15 limit 14 to ed=20220328-20250617 62

16 (202203* or 202204* or 202205* or 202206* or 202207* or 202208* or 202209* or 20221* or 2023* or 2024* or 2025*).dt,ez,da. 5613407

17 14 and 16 74

18 15 or 17 75

<https://ovidsp.ovid.com/ovidweb.cgi?T=JS&NEWS=N&PAGE=main&SHAREDSEARCHID=7SL2lxgVgidvAisANfInrEeTfufMH64AQ3oLWPO9jOTCFaao46qt31f1SLxLvRQTA>

**Central**

1 exp Infant/ 44719

2 exp Hyperbilirubinemia, Neonatal/ 429

3 neonat*.tw. 28378

4 (infant* or infanc*).tw. 49352

5 newborn*.tw. 12607

6 1 or 2 or 3 or 4 or 5 88963

7 Phototherapy/ 1114

8 phototherap*.tw. 2835

9 light therap*.tw. 1503

10 blue light.tw. 845

11 7 or 8 or 9 or 10 5213

12 exp Neoplasms/ 127096

13 exp biopsy/ 7769

14 (cancer* or neoplas* or carcino* or adenocarcinoma* or malignanc* or tumor* or tumour* or malignan* or metasta* or sarcoma* or chromatid exchange* or leukemia* or leukaemia* or melanoma* or biops*).tw. 299839

15 12 or 13 or 14 320711

16 6 and 11 and 15 13

17 (2022-03* or 2022-04* or 2022-05* or 2022-06* or 2022-07* or 2022-08* or 2022-09* or 2022-1* or 2023* or 2024* or 2025*).dl. 457070

18 16 and 17 2

<https://ovidsp.ovid.com/ovidweb.cgi?T=JS&NEWS=N&PAGE=main&SHAREDSEARCHID=1j02jSkdC7458Cx6XYqbtWu2H6FaMPPgUEKUMe12DRpj2DYEdVfRQj1pSBW6lgYbc>

**CINAHL**

| S19 | S17 AND S18 | View Results (10) |
| --- | --- | --- |
| S18 | EM 20220328- | View Results (842,727) |
| S17 | S6 AND S12 AND S16 | View Results (58) |
| S16 | S13 OR S14 OR S15 | View Results (1,030,684) |
| S15 | TI ( (cancer* or neoplas* or carcino* or adenocarcinoma* or malignanc* or tumor* or tumour* or malignan* or metasta* or sarcoma* or chromatid exchange* or leukemia* or leukaemia* or melanoma* or biops*) ) OR AB ( (cancer* or neoplas* or carcino* or adenocarcinoma* or malignanc* or tumor* or tumour* or malignan* or metasta* or sarcoma* or chromatid exchange* or leukemia* or leukaemia* or melanoma* or biops*) ) | View Results (851,686) |
| S14 | (MH "Biopsy+") | View Results (55,402) |
| S13 | (MH "Neoplasms+") | View Results (680,855) |
| S12 | S7 OR S8 OR S9 OR S10 OR S11 | View Results (8,663) |
| S11 | TI "photoradiation therap*" OR AB "photoradiation therap*" | View Results (6) |
| S10 | TI "blue light" OR AB "blue light" | View Results (666) |
| S9 | TI "light therap*" OR AB "light therap*" | View Results (843) |
| S8 | TI phototherap* OR AB phototherap* | View Results (2,256) |
| S7 | (MH "Phototherapy+") | View Results (6,873) |
| S6 | S1 OR S2 OR S3 OR S4 OR S5 | View Results (388,232) |
| S5 | TI newborn* OR AB newborn* | View Results (39,647) |
| S4 | TI ( (infant* or infanc*) ) OR AB ( (infant* or infanc*) ) | View Results (142,582) |
| S3 | TI neonat* OR AB neonat* | View Results (88,620) |
| S2 | (MH "Hyperbilirubinemia, Neonatal+") | View Results (1,943) |
| S1 | (MH "Infant+") | View Results (298,666) |

**Embase**

1 exp infant/ 1459392

2 neonatal hyperbilirubinemia/ 2059

3 neonat*.tw,kf. 485181

4 (infant* or infanc*).tw,kf. 762141

5 newborn*.tw,kf. 292839

6 1 or 2 or 3 or 4 or 5 1920335

7 exp phototherapy/ 136247

8 exp phototherapy device/ 3458

9 phototherap*.tw,kf. 20364

10 light therap*.tw,kf. 5431

11 "blue light".tw,kf. 15589

12 photoradiation therap*.tw,kf. 198

13 7 or 8 or 9 or 10 or 11 or 12 155371

14 exp neoplasm/ 7029284

15 exp biopsy/ 1076801

16 (cancer* or neoplas* or carcino* or adenocarcinoma* or malignanc* or tumor* or tumour* or malignan* or metasta* or sarcoma* or chromatid exchange* or leukemia* or leukaemia* or melanoma* or biops*).tw,kf. 7420456

17 14 or 15 or 16 9018121

18 6 and 13 and 17 1521

19 limit 18 to embase 1218

20 limit 19 to dc=20220328-20250617 278

<https://myaccess.library.utoronto.ca/login?url=http://ovidsp.ovid.com/ovidweb.cgi?T=JS&NEWS=N&PAGE=main&SHAREDSEARCHID=22RLAhnoRTXUfrp5KsK30nGGgOedgd3ld3HWbOh9U42zdxva80ARMQDwJniYPPrbx>

**Scopus**

( ( ( TITLE-ABS-KEY ( neonat* ) ) OR ( TITLE-ABS-KEY ( ( infant* OR infanc* ) ) ) OR ( TITLE-ABS-KEY ( newborn* ) ) ) AND ( ( TITLE-ABS-KEY ( phototherap* ) ) OR ( TITLE-ABS-KEY ( "light therap*" ) ) OR ( TITLE-ABS-KEY ( "blue light" ) ) ) AND ( TITLE-ABS-KEY ( cancer* OR neoplas* OR carcino* OR adenocarcinoma* OR malignanc* OR tumor* OR tumour* OR malignan* OR metasta* OR sarcoma* OR chromatid AND exchange* OR leukemia* OR leukaemia* OR melanoma* OR biops* ) ) ) AND ( ORIG-LOAD-DATE > 20220328 )
